# Long-term health-related quality of life among adolescent and young adult breast cancer survivors

**DOI:** 10.1007/s11136-025-03914-1

**Published:** 2025-02-21

**Authors:** Noelle J. M. C. Vrancken Peeters, Roos Kerklaan, Carla Vlooswijk, Rhodé M. Bijlsma, Suzanne E. J. Kaal, Jacqueline M. Tromp, Monique E. M. M. Bos, Tom van der Hulle, Maaike de Boer, Janine Nuver, Mathilde C. M. Kouwenhoven, Winette T. A. van der Graaf, Olga Husson

**Affiliations:** 1 Department of Medical Oncology, Netherlands Cancer Institute-Antoni Van Leeuwenhoek, 1066 CX Amsterdam, The Netherlands; 2 Department of Plastic and Reconstructive Surgery, Erasmus MC Cancer Institute, Erasmus University Medical Centre, 3015 GD Rotterdam, The Netherlands; 3 Research and Development, Netherlands Comprehensive Cancer Organisation, 3511 CV Utrecht, The Netherlands; 4 Department of Medical Oncology, University Medical Centre, 3584 CX Utrecht, The Netherlands; 5 Department of Medical Oncology, Radboud University Medical Centre, 6525 GA Nijmegen, The Netherlands; 6 Department of Medical Oncology, Amsterdam University Medical Centres, 1105 AZ Amsterdam, The Netherlands; 7 Department of Medical Oncology, Erasmus MC Cancer Institute, Erasmus University Medical Centre, 3015 GD Rotterdam, The Netherlands; 8 Department of Medical Oncology, Leiden University Medical Centre, 2333 ZA Leiden, The Netherlands; 9 Department of Medical Oncology, Maastricht University Medical Center, 6202 AZ Maastricht, The Netherlands; 10 Department of Medical Oncology, University Medical Centre Groningen, 9713 GZ Groningen, The Netherlands; 11 Department of Neurology, Amsterdam UMC, Amsterdam University Medical Centres, Location VUmc, 1081 HV Amsterdam, The Netherlands; 12 Department of Psychosocial Research and Epidemiology, Netherlands Cancer Institute, 1066 CX Amsterdam, The Netherlands; 13 Department of Surgical Oncology, Erasmus MC Cancer Institute, Erasmus University Medical Centre, 3015 GD Rotterdam, The Netherlands

**Keywords:** Adolescents and young adults (AYAs), Breast cancer, Health-related quality of life (HRQoL), Survivorship

## Abstract

**Purpose:**

As the prognosis for adolescents and young adults (AYAs) with breast cancer has improved, long-term health-related quality of life (HRQoL) has become increasingly important. This study aimed to analyze the long-term HRQoL of AYA breast cancer survivors compared to an age-matched normative population and to identify factors associated with HRQoL.

**Methods:**

Secondary analyses were conducted using data from the SURVAYA study. The European Organization for Research and Treatment of Cancer Quality of Life Questionnaire Core-30 (EORTC QLQ-C30) was used to assess HRQoL. The Mann–Whitney U test was used to compare HRQoL scores of AYA breast cancer survivors with those of the normative population (n = 409). Linear regression models were constructed to identify patient and treatment characteristics associated with HRQoL.

**Results:**

A total of 944 female AYA breast cancer survivors were included, with a median age of 36.0 years and a median follow-up of 12.2 years. AYA breast cancer survivors scored significantly lower on five functional scales: physical, role, emotional, cognitive, and social, and higher on five symptom scales: fatigue, pain, dyspnea, insomnia, and financial impact compared to the normative population. Being in a relationship, having a positive body image, and adaptive coping were positively associated with HRQoL, while older age, chemotherapy, unemployment, and maladaptive coping were negatively associated.

**Conclusion:**

AYA breast cancer survivors experience significantly compromised long-term HRQoL compared to an age-matched normative population. These results highlight the need for tailored follow-up care and long-term support, as well as the importance of shared decision-making about the benefits and risks of treatments before initiation.

**Supplementary Information:**

The online version contains supplementary material available at 10.1007/s11136-025-03914-1.

## Introduction

Adolescents and young adults (AYAs) are defined as individuals aged between 15 and 39 years at the time of their initial cancer diagnosis, according to the US National Cancer Institute [1]. However, the AYA age range may be defined differently in other healthcare systems [2]. In the Netherlands, children (0–18 years) receive treatment and support centrally in a pediatric oncology center, while adults (≥ 18 years) are treated in general and academic hospitals in the whole country. Due to this dichotomization, AYAs in the Netherlands are defined as individuals aged from 18 to 39 years at their initial cancer diagnosis [3].

Currently, the incidence of cancers in AYAs is increasing [4, 5]. AYAs are recognized as a unique population within oncology. AYAs have a long life ahead because of their prolonged survival period, with an overall 5-year relative survival rate of over 80% and 20-year relative survival of 74% [6, 7]. But most importantly, AYAs express age-specific needs as they find themselves in a developmental life phase. AYA cancer survivors are impacted by both physical issues, such as possible infertility, as well as more psychosocial matters, such as the interruption of education or career development, problems with sexuality and intimacy, and difficulties in building and maintaining mature relationships [8–10]. Furthermore, AYAs encounter more delays in their diagnosis and treatment due to failing to recognize or acknowledge the importance of their symptoms [11, 12].

Breast cancer is the most common type of cancer among female AYAs worldwide [5, 13]. Despite the increasing incidence of breast cancer among AYAs, there is a positive trend in the prognosis due to advances in various treatment modalities [4, 10, 14–16]. Although this rise in life expectancy is promising, AYA breast cancer survivors must live with the possible long-term adverse effects of breast cancer and its treatment, which can have a significant impact on their overall health-related quality of life (HRQoL) [17–19].

To date, literature on long-term HRQoL of AYA breast cancer survivors is scarce. Previous research focused either on older breast cancer survivors or on AYA cancer in general [20–24]. Yet HRQoL impairments differ by age, and breast cancer is a unique type of cancer as the breasts are regarded as one of the most important aspects of female identity and sexuality. As a result, breast cancer survivors often report lower HRQoL compared to other cancer survivors [25–28].

The primary aim of this study was to analyze the HRQoL of AYA breast cancer survivors and compare the HRQoL of AYA breast cancer survivors with that of an age-matched normative population to identify any persistent long-term HRQoL issues associated with their cancer diagnosis and treatment. The secondary aim of this study was to identify factors associated with the HRQoL of AYA breast cancer survivors. The results of this study will provide healthcare professionals with valuable insights into areas where additional long-term support may be needed to improve HRQoL of AYA breast cancer survivors in the future.

## Methods

### Data collection

The SURVAYA (health-related quality of life and late effects among SURVivors of cancer in Adolescence and Young Adulthood) study data was used for secondary analyses [3]. The SURVAYA study is a retrospective, population-based, observational, cross-sectional cohort questionnaire study conducted among long-term (5–20 years post-diagnosis) AYA cancer survivors (18–39 years at the time of diagnosis) in the Netherlands. AYA cancer survivors were invited to participate in the SURVAYA study from the involved cancer centers, including eight university medical centers and the Netherlands Cancer Institute (NCI), using the Netherlands Cancer Registry (NCR) [29]. The NCR collects detailed data on cancer patients in the Netherlands, including disease and treatment characteristics. Patient-reported questionnaire data of the SURVAYA study were collected using the PROFILES (Patient-Reported Outcomes Following Initial Treatment and Long-term Evaluation of Survivorship) registry and merged with the NCR data at the end of the study [30]. More detailed information concerning the SURVAYA study has previously been published elsewhere [3].

### Normative population

An age-matched normative population, consisting of women from the Dutch general population without a cancer diagnosis, was obtained from CentERdata, a research institute at Tilburg University. CentERdata has an online household panel comprising more than 2000 Dutch households and is representative of the Dutch-speaking population in the Netherlands [31]. The normative data used for this study were collected in 2017.

The normative population was matched to the AYA breast cancer survivors using a frequency matching method with age at the time of filling out the EORTC QLQ-C30 strata (20–35 years, 35–50 years, and 50–65 years) [32]. A total of 409 panel members were matched to 944 AYA breast cancer survivors.

### HRQoL

HRQoL was assessed using the Dutch version of the European Organization for Research and Treatment of Cancer Quality of Life Questionnaire Core-30 (EORTC QLQ-C30, version 3) [33, 34]. The EORTC QLQ-C30 consists of 30 questions divided over 15 scales, including five functional scales (physical functioning, role functioning, cognitive functioning, emotional functioning, and social functioning), eight symptom scales (fatigue, nausea and vomiting, pain, dyspnea, insomnia, appetite loss, constipation, and diarrhea), a financial impact scale and a quality of life (QoL) scale. A four-point ordinal Likert scale was used for the functional, financial impact, and symptom scales ranging from 1 = not at all, 2 = a little, 3 = quite a bit, to 4 = very much. For the QoL scale, an ordinal scale ranging from 1 = very poor to 7 = excellent was used. All scales and item scores were linearly transformed into numeric scores (0–100). For the functional scales and the QoL scale, a higher score represents a better level of functioning. For the symptom scales and the financial impact scale, a higher score represents a higher level of symptoms/difficulties [33, 35]. A difference of 5 points was considered clinically relevant [36].

All domains of the EORTC QLQ-C30 were included in the analysis to encompass all aspects of survivorship. The hypothesis posited that while acute symptoms like nausea or dyspnea would not significantly differ between long-term AYA breast cancer survivors and the normative population, longer-term symptoms such as fatigue and cognitive issues would exhibit significant differences.

### Covariates

Patient demographics (at the time of questionnaire completion), treatment modality, and tumor characteristics were obtained from the SURVAYA study and the NCR. Variables included in the analysis were selected based on literature and encompassed the following: age at diagnosis, time since diagnosis, body mass index (BMI) categorized as overweight (BMI > 25) and normal weight (BMI < 25), educational level, relationship status, employment status, chemotherapy, radiotherapy, hormonal therapy, breast and axillary surgery including breast-conserving surgery (BCS) with or without axillary lymph node dissection (ALND) and mastectomy with or without ALND, body image and coping mechanisms (maladaptive and adaptive) [26, 37–39].

#### Body image

Body image was assessed using the EORTC QLQ-SURV100, a questionnaire specifically developed for disease-free cancer survivors, at least one year post-treatment, covering the long-term effects of a cancer diagnosis and treatment. Body image is a functional scale consisting of two single items; “Have you felt older than your age?” and “Have you been dissatisfied with your physical appearance?”. A higher score on this scale indicates a more positive body image [3, 40].

#### The cognitive emotion regulation questionnaire (CERQ)

The CERQ was used to measure the cognitive coping mechanisms [41]. The CERQ consists of nine scales: self-blame, other-blame, rumination, catastrophizing, positive refocusing, planning, positive reappraisal, putting into perspective, and acceptance. Each scale of the full CERQ contains four items. In this study, a condensed version utilizing two items per scale was used. For each item, the answering options ranged from 1 [(almost) never] to 5 [(almost) always]. To calculate the total scale score, the scores of the two relevant items are summed, resulting in a range from 2 to 10. A higher score represents more usage of that specific cognitive emotion regulation strategy. A maladaptive scale based on self-blame, other-blame, rumination, and catastrophizing, and an adaptive scale based on positive refocusing, planning, positive reappraisal, putting into perspective, and acceptance were constructed. The maladaptive scale ranges from 8 to 40, while the adaptive scale ranges from 10 to 50 [41, 42].

### Data analysis

Descriptive statistics were used to describe patient, tumor, and treatment characteristics, as well as EORTC QLQ-C30 scores. The Mann–Whitney-U test was used to compare the HRQoL between AYA breast cancer survivors and the normative population.

Multiple linear regression models were used to identify patient and treatment characteristics associated with HRQoL of AYA breast cancer survivors, focusing on the EORTC QLQ-C30 scales that showed significant differences between AYA breast cancer survivors and the normative population (physical functioning, role functioning, emotional functioning, cognitive functioning, social functioning, fatigue, pain, dyspnea, insomnia, and financial impact). Variables included in the multiple linear regression models were age at diagnosis, time since diagnosis, BMI, educational level, relationship status, employment status, chemotherapy, radiotherapy, hormonal therapy, breast and axillary surgery, body image, maladaptive coping, and adaptive coping. To avoid multicollinearity, the tumor stage was not included. Additionally, for the regression models of the functional scales, fatigue, pain, and insomnia from the symptom scales of the EORTC QLQ-C30 were also included.

A variance inflation factor (VIF) was calculated to check for multicollinearity. A VIF with a score < 5 was considered acceptable for analysis [43]. Normality and homoscedasticity of the residuals were tested with residual plots. A two-sided p-value of 0.05 or less was considered statistically significant. All statistical analyses were performed using R statistical software (version 4.4.3) [44].

#### Non-responder analysis

A non-responder analysis was conducted to assess differences in baseline characteristics between responders and non-responders. Non-responders were defined as AYA breast cancer survivors who did not answer any questions of the EORTC QLQ-C30. Numeric variables were analyzed using an unpaired Student’s t-test, and categorical variables were examined using chi-squared tests. A complete case analysis was performed due to the limited amount of missing data.

## Results

### Patient characteristics

A total of 944 female AYA breast cancer survivors were included in the SURVAYA study. The median age at diagnosis was 36.0 years, ranging from 18.0 to 39.0 years, and the median time since diagnosis was 12.2 years, ranging from 4.41 to 21.6 years. Almost all AYA breast cancer survivors were diagnosed with tumor stage 1 (35.8%) and tumor stage 2 (47,4%). Most survivors underwent chemotherapy (85.5%), radiotherapy (77.3%), and hormonal therapy (50.7%). Additionally, BCS without ALND was the most common treatment AYA breast cancer survivors received (39.1%, Table 1).

**Table 1:** Baseline characteristics of AYA breast cancer survivors

Characteristics	AYA breast cancer survivors (n = 944)
Age at diagnosis (years)	
Mean (SD)	34.7 (3.85)
Median [Min, Max]	36.0 [18.0, 39.0]
Time since diagnosis (years)	
Mean (SD)	12.2 (4.52)
Median [Min, Max]	12.2 [4.41, 21.6]
Age at time of filling out EORTC QLQ-C30 (years)	
Mean (SD)	47.5 (6.13)
Median [Min, Max]	47.8 [23.7, 60.0]
BMI, n (%)	
Normal weight^a^	573 (60.7)
Overweight^a^	354 (37.5)
Missing	17 (1.8)
Educational level, n (%)	
Low, no primary school	5 (0.5)
Intermediate, secondary education	377 (39.9)
High, college/university	562 (59.5)
Relationship status, n (%)	
Registered partnership/married	545 (57.7)
Relationship	247 (26.2)
Single	147 (15.6)
Missing	5 (0.5)
Employment status, n (%)	
Employed	664 (70.3)
Self employed	129 (13.7)
Unemployed	151 (16.0)
Tumor stage, n (%)	
1	338 (35.8)
2	447 (47.4)
3	153 (16.2)
4	6 (0.6)
Chemotherapy, n (%)	
Yes	807 (85.5)
No	137 (14.5)
Radiotherapy, n (%)	
Yes	730 (77.3)
No	214 (22.7)
Hormonal therapy, n (%)	
Yes	479 (50.7)
No	465 (49.3)
Targeted therapy, n (%)	
Yes	173 (18.3)
No	771 (81.7)
Breast and axillary surgery, n (%)	
BCS	369 (39.1)
BCS + ALND	133 (14.1)
Mastectomy	216 (22.9)
Mastectomy + ALND	219 (23.2)
Missing	7 (0.7)
Body image	
Mean (SD)	72.0 (25.7)
Median [Min, Max]	83.3 [0, 100]
Missing	59 (6.3)
Maladaptive scale	
Mean (SD)	13.1 (3.60)
Median [Min, Max]	13.0 [8.00, 27.0]
Missing	72 (7.6)
Adaptive scale	
Mean (SD)	29.9 (7.18)
Median [Min, Max]	30.0 [10.0, 50.0]
Missing	75 (7.9)

*ALND* axillary lymph node dissection, *BCS* breast-conserving surgery, *BMI* body mass index, *EORTC QLQ-C30* European Organization for Research and Treatment of Cancer Quality of Life Questionnaire Core-30, SD; standard deviation

^a^BMI was categorized as overweight (BMI > 25) and normal weight (BMI < 25)

The baseline characteristics of the normative population (n = 409) can be found in Appendix Table 4.

No significant differences in the baseline characteristics between responders and non-responders (n = 57, 6%) were observed (p > 0.05, Appendix Table 5).

### EORTC QLQ-C30 scores of AYA breast cancer survivors compared to the normative population

**Fig. 1:**
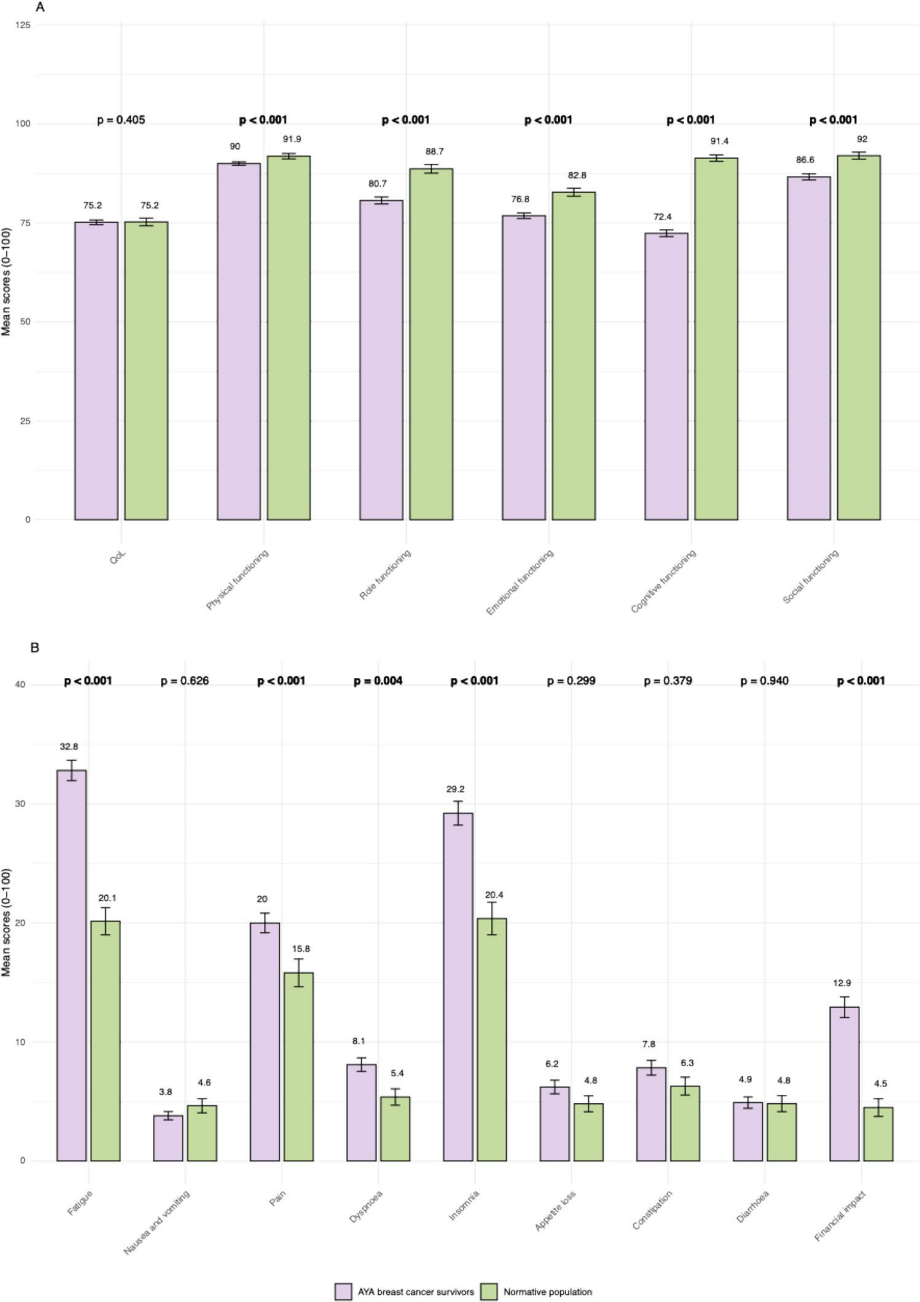
Bar charts of the mean scores of EORTC QLQ-C30 functional scales (**a**) and symptom scales (**b**) for AYA breast cancer survivors (purple) and the normative population (green). A higher score on the functional scales represents a higher level of functioning, and a higher score on the symptom scales represents a higher level of symptoms. Abbreviations: *AYA* Adolescent and Young Adult, *EORTC QLQ-C30* European Organization for Research and Treatment of Cancer Quality of Life Questionnaire Core-30, *QoL* quality of life. Error bars represent the standard error of the mean. Bold indicates P-value < 0.05

AYA breast cancer survivors scored significantly lower on physical functioning, role functioning, emotional functioning, cognitive functioning, and social functioning (p < 0.001). The largest difference between the groups on the functional scales was found in cognitive functioning, with AYA breast cancer survivors having a mean score of 72.4 compared to 91.4 in the normative population (p < 0.001). On the role functioning, emotional functioning, cognitive functioning, and social functioning scales, a difference of 5 points or more was observed (Fig. 1).

On the symptom scales, AYA breast cancer survivors scored significantly higher on fatigue (p < 0.001), pain (p < 0.001), dyspnea (p = 0.004), insomnia (p < 0.001), and financial impact (p < 0.001). The largest difference between the groups was observed in the fatigue scale, where AYA breast cancer survivors had a mean score of 32.8 compared to 20.1 in the normative population (p < 0.001). Differences of 5 points or more were observed on the fatigue, insomnia, and financial impact scales (Fig. 1).

The standard deviations (SD), medians, and ranges of the EORTC QLQ-C30 scales are provided in Appendix Table 6.

### Factors associated with long-term HRQoL of AYA breast cancer survivors

Being in a relationship or being married compared to being single and having an adaptive coping mechanism were positively associated with HRQoL. Additionally, a higher body image score was positively associated with HRQoL on all functional and symptom scales of the EORTC QLQ-C30 (Tables 2 and 3).

**Table 2:** Multiple linear regression models of the functional scales of the EORTC QLQ-C30 among the AYA breast cancer survivors

Variables	Physical functioning		Role functioning		Emotional functioning		Cognitive functioning		Social functioning	
	Estimates	p	Estimates	p	Estimates	p	Estimates	p	Estimates	p
Age at diagnosis	− 0.24	**0.011**	− 0.21	0.200	0.13	0.380	− 0.43	**0.030**	− 0.21	0.199
Time since diagnosis	0.07	0.422	0.00	0.992	− 0.11	0.379	0.31	0.074	0.08	0.569
BMI										
Normal weight	*Reference*		*Reference*		*Reference*		*Reference*		*Reference*	
Overweight	− 1.77	**0.020**	0.52	0.692	1.56	0.174	3.07	**0.050**	1.00	0.442
Educational level										
Intermediate, secondary education	*Reference*		*Reference*		*Reference*		*Reference*		*Reference*	
Low, no primary school	2.57	0.666	6.17	0.546	6.49	0.469	− 8.48	0.489	6.10	0.548
High, college/university	2.03	**0.006**	− 3.47	**0.007**	0.42	0.707	5.61	** < 0.001**	− 3.45	**0.007**
Relationship status										
Single	*Reference*		*Reference*		*Reference*		*Reference*		*Reference*	
Married/registered partnership	1.43	0.152	3.27	0.057	− 2.01	0.184	− 1.15	0.577	1.81	0.292
Relationship	2.65	**0.020**	4.48	**0.022**	− 0.59	0.729	0.29	0.903	3.74	0.055
Employment status										
Employed	*Reference*		*Reference*		*Reference*		*Reference*		*Reference*	
Self employed	− 1.64	0.117	− 2.37	0.187	− 1.37	0.385	− 1.20	0.577	− 0.53	0.769
Unemployed	− 5.38	** < 0.001**	− 8.34	** < 0.001**	− 0.51	0.744	− 3.90	0.068	− 6.43	** < 0.001**
Chemotherapy										
No	*Reference*		*Reference*		*Reference*		*Reference*		*Reference*	
Yes	− 1.27	0.242	− 3.14	0.091	− 0.63	0.698	− 4.89	**0.028**	− 3.74	**0.043**
Radiotherapy										
No	*Reference*		*Reference*		*Reference*		*Reference*		*Reference*	
Yes	0.19	0.868	1.09	0.573	− 0.32	0.849	2.02	0.381	0.90	0.638
Hormonal therapy										
No	*Reference*		*Reference*		*Reference*		*Reference*		*Reference*	
Yes	− 1.26	0.092	− 1.52	0.235	0.67	0.550	− 0.86	0.574	− 1.41	0.268
Breast and axillary surgery										
BCS	*Reference*		*Reference*		*Reference*		*Reference*		*Reference*	
BCS + ALND	− 0.03	0.978	− 0.99	0.604	− 0.59	0.724	0.73	0.749	− 1.03	0.587
Mastectomy	1.31	0.278	2.20	0.289	− 2.08	0.254	− 0.50	0.840	− 0.52	0.802
Mastectomy + ALND	− 0.94	0.344	− 0.33	0.844	1.87	0.210	2.40	0.238	0.65	0.699
Body image	0.07	** < 0.001**	0.11	** < 0.001**	0.21	** < 0.001**	0.19	** < 0.001**	0.16	** < 0.001**
Maladaptive scale	0.12	0.269	− 0.01	0.969	− 1.63	** < 0.001**	− 0.20	0.365	− 0.52	**0.004**
Adaptive scale	0.08	0.087	0.03	0.688	0.21	**0.005**	− 0.07	0.475	0.02	0.814
Fatigue	− 0.16	** < 0.001**	− 0.30	** < 0.001**	− 0.20	** < 0.001**	− 0.33	** < 0.001**	− 0.23	** < 0.001**
Pain	− 0.16	** < 0.001**	− 0.46	** < 0.001**	− 0.02	0.415	− 0.05	0.143	− 0.23	** < 0.001**
Insomnia	0.01	0.477	− 0.02	0.414	− 0.15	** < 0.001**	− 0.09	**0.001**	− 0.06	**0.004**
Observations	851		851		851		851		850	
R^2^/R^2^ adjusted	0.455/0.441		0.560/0.549		0.483/0.470		0.350/0.333		0.449/0.435	

*ALND* axillary lymph node dissection, *BCS* breast-conserving surgery, *BMI* body mass index

Bold indicates P < 0.05

**Table 3:** Multiple linear regression models of the symptom scales of the EORTC QLQ-C30 among the AYA breast cancer survivors

Variables	Fatigue		Pain		Dyspnea		Insomnia		Financial impact	
	Estimates	p	Estimates	p	Estimates	p	Estimates	p	Estimates	p
Age at diagnosis	− 0.14	0.476	0.53	**0.006**	− 0.08	0.568	0.8	**0.002**	0.07	0.739
Time since diagnosis	− 0.38	**0.031**	0	0.995	− 0.08	0.564	0.06	0.789	− 0.1	0.584
BMI										
Normal weight	*Reference*		*Reference*		*Reference*		*Reference*		*Reference*	
Overweight	− 3.92	**0.015**	− 2.4	0.122	1.46	0.221	− 1.95	0.352	− 1.18	0.477
Educational level										
Intermediate, secondary education	*Reference*		*Reference*		*Reference*		*Reference*		*Reference*	
Low, no primary school	− 22.86	0.069	− 6.65	0.584	− 8.16	0.383	− 3.53	0.83	20.78	0.11
High, college/university	1.01	0.517	− 2.67	0.078	1.41	0.225	− 2.56	0.21	− 1.43	0.375
Relationship status										
Single	*Reference*		*Reference*		*Reference*		*Reference*		*Reference*	
Married/registered partnership	− 4.81	**0.023**	− 1.9	0.353	− 5.03	**0.001**	0.61	0.826	− 7.84	** < 0.001**
Relationship	− 4.76	**0.048**	0.79	0.733	− 4.72	**0.008**	1.77	0.573	− 7.01	**0.005**
*Employment status*										
Employed	*Reference*		*Reference*		*Reference*		*Reference*		*Reference*	
Self employed	1.5	0.497	2.29	0.283	1.59	0.333	3.32	0.25	4.93	**0.031**
Unemployed	7.93	** < 0.001**	7.33	** < 0.001**	2.77	0.086	2.75	0.331	18.16	** < 0.001**
Chemotherapy										
No	*Reference*		*Reference*		*Reference*		*Reference*		*Reference*	
Yes	1.71	0.455	0.92	0.677	0.51	0.767	− 1.82	0.542	0.44	0.851
Radiotherapy										
No	*Reference*		*Reference*		*Reference*		*Reference*		*Reference*	
Yes	− 0.59	0.804	− 1.07	0.639	1.16	0.51	4.91	0.113	− 2.38	0.328
Hormonal therapy										
No	*Reference*		*Reference*		*Reference*		*Reference*		*Reference*	
Yes	0.18	0.907	− 0.42	0.785	0.66	0.573	1.18	0.568	0.32	0.847
Breast and axillary surgery										
BCS	*Reference*		*Reference*		*Reference*		*Reference*		*Reference*	
BCS + ALND	− 0.22	0.925	− 2.57	0.257	3.47	**0.047**	− 0.46	0.88	− 0.36	0.882
Mastectomy	− 2.5	0.325	− 1.95	0.428	− 1.37	0.47	1.79	0.591	− 1.04	0.693
Mastectomy + ALND	0.99	0.636	− 2.32	0.251	0.24	0.876	0.27	0.921	− 0.68	0.753
Body image	− 0.45	** < 0.001**	− 0.45	** < 0.001**	− 0.18	** < 0.001**	− 0.35	** < 0.001**	− 0.3	** < 0.001**
Maladaptive scale	0.68	**0.002**	0.15	0.468	0.07	0.665	0.93	**0.001**	1.08	** < 0.001**
Adaptive scale	0.08	0.435	0.16	0.122	0.04	0.629	− 0.05	0.728	0.03	0.754
Observations	852		852		852		851		851	
R^2^/R^2^ adjusted	0.300/0.285		0.281/0.266		0.122/0.103		0.137/0.119		0.267/0.251	

*ALND* axillary lymph node dissection, *BCS* breast-conserving surgery, *BMI* body mass index

Bold indicates P < 0.05

An older age at diagnosis, being unemployed in comparison to being employed, having received chemotherapy, and having a maladaptive coping mechanism were negatively associated with HRQoL (Tables 2 and 3).

Being overweight compared to having a normal weight and having a higher educational level compared to an intermediate educational level had both positive and negative associations with different aspects of HRQoL. Overweight AYA breast cancer survivors scored lower on physical functioning and fatigue and higher on cognitive functioning. AYA breast cancer survivors with a high educational level scored higher on physical functioning and cognitive functioning and lower on role functioning and social functioning (Tables 2 and 3). Moreover, fatigue, pain, and insomnia were negatively associated with HRQoL (functional scales, Table 2).

## Discussion

AYA breast cancer survivors, with a median follow-up of 12.2 years, had significantly lower HRQoL than the matched normative population. AYA breast cancer survivors scored significantly lower on functional scales of the EORTC QLQ-C30 (physical, role, emotional, cognitive, and social) and higher on symptom scales (fatigue, pain, dyspnea, insomnia, and financial impact). Most differences in HRQoL were not only statistically significant but also clinically meaningful, particularly in cognitive functioning and fatigue [36]. Additionally, age at diagnosis, BMI, relationship status, educational level, employment status, chemotherapy, body image, and coping mechanisms were associated with HRQoL of AYA breast cancer survivors.

### Functioning

The significant impairment in cognitive functioning among AYA breast cancer survivors compared to the normative population could be attributed to the intensive chemotherapy and long-term hormonal therapy, including ovarian ablation often required for treating the aggressive types of cancers in this group, which increases the likelihood of developing cognitive issues, such as problems with thinking, memory, and concentration [45–58]. Previous research found that self-reported cognitive problems were highest for breast cancer patients who received both chemotherapy and hormone therapy (OR = 6.33, 95% CI = 4.21, 9.54), followed by those who received only chemotherapy (OR = 5.63, 95% CI = 3.52, 9.00), and those who received only hormone therapy (OR = 1.64, 95% CI = 1.15, 2.33), compared with those reporting neither treatment [57]. Similarly, Mandelblatt et al. identified a significantly higher likelihood of accelerated cognitive decline among survivors receiving chemotherapy (with and without hormonal therapy) compared to those receiving only hormonal therapy (OR = 2.1, 95% CI = 1.3–3.5) [58]. Moreover, Schagen et al. have shown that breast cancer patients (mean age 47.1 years) treated with chemotherapy have a significantly higher risk of late cognitive impairment compared to those without chemotherapy (OR = 6.4, 95% CI = 1.5–27.6) [55]. However, Dijkshoorn et al. demonstrated that one in four breast cancer patients had cognitive impairment even before starting anticancer treatment, suggesting that psychological factors such as post-traumatic stress disorder (PTSD), which is common after a cancer diagnosis, can also play an important role [54, 59]. The linear regression analysis in the current study confirmed a negative association between chemotherapy and cognitive functioning, while no association with hormonal therapy was found. This might be explained by the fact that cognitive functioning in the current study was assessed using only the two EORTC QLQ-C30 items. Research suggests that hormonal therapy may have more pronounced effects on specific cognitive domains, such as verbal learning/memory, which were not comprehensively assessed in this study [47–50].

Furthermore, previous research among older breast cancer patients with a shorter follow-up duration showed a significant decrease in role, emotional, and social functioning scores compared to the general population; however, no statistically significant difference in physical functioning was found [60, 61]. While these findings align with those of the current study, this study also identified a significant decrease in physical functioning. This discrepancy may be explained by the fact that older cancer survivors tend to adjust their health perceptions more easily, whereas younger survivors generally have higher expectations for their physical abilities [62].

### Symptoms

AYA breast cancer survivors reported statistically and clinically significant higher levels of fatigue and insomnia compared to the normative population. It is known that cancer-related fatigue is common among cancer patients and is considered one of the most burdensome symptoms experienced during and after treatment [63]. Arndt et al. demonstrated that fatigue and insomnia were more common in breast cancer patients (mean age 58.2 years) three years after diagnosis compared to the general population [61]. Similarly, a study with a six-year follow-up period reported that fatigue and insomnia were common in breast cancer survivors and associated with radiotherapy and hormonal therapy [64]. The current study, with a longer follow-up of 12 years, did not find any association between these symptoms and treatment characteristics. This discrepancy could be attributed to the possibility that the more immediate effects of treatment diminish over time.

AYA breast cancer survivors also reported significantly higher, yet not clinically significant, pain and dyspnea scores. The prevalence of post-mastectomy pain syndrome, chronic pain in the breast/chest wall, axilla, or arm often of neurological origin after breast surgery, is known to be high in breast cancer survivors. A young age is an independent risk factor and may explain the high pain scores observed in this study [65–67]. Moreover, the higher dyspnea scores could be explained by the pulmonary effects of radiotherapy, as breast radiotherapy can lead to lung damage such as fibrosis [68, 69]. However, the current study did not find a direct association between dyspnea and radiotherapy. Instead, relationship status and body image were associated, suggesting that the psychosocial state of AYA breast cancer survivors could also influence the reported dyspnea.

Nausea and vomiting, appetite loss, constipation, and diarrhea symptoms were less common in the current study, which might be explained by the fact that these symptoms are more likely to be directly related to breast cancer treatment and may diminish over time [70].

Lastly, fatigue, pain, and insomnia were found to negatively impact all functional scales of the EORTC QLQ-C30, aligning with findings from De Ligt et al., which showed that health symptoms adversely affect daily functioning in early-stage breast cancer survivors (median age 62.2 ± 10.9 years) [60]. Additionally, Arndt et al. found that fatigue was the strongest predictor for functioning and overall QoL compared to other symptoms in breast cancer patients one year after diagnosis [71].

### Financial impact

AYA breast cancer survivors reported statistically and clinically significant higher financial impact scores than the normative population. This difference can be explained by the fact that AYA cancer survivors often miss critical developmental milestones, such as completing their education or starting their first job, which is likely to impact their financial future [8–10, 72]. Additionally, unemployment was found to impact HRQoL negatively. The findings align with previous research showing that AYA cancer survivors are more frequently unemployed than matched controls and that a cancer diagnosis at a young age significantly impacts income levels [72, 73]. This highlights the need for financial support for AYA cancer survivors. Currently, eight members of the EU have implemented ‘Rights To Be Forgotten’ laws for cancer survivors to protect them from financial toxicity. There is an ongoing discussion about whether this period should be shortened for AYA cancer survivors [74].

### Overall QoL

Literature on older patients demonstrated that although long-term breast cancer survivors scored lower on the functional and higher on most symptom scales of the EORTC QLQ-C30 compared to the normative population, there was most often no significant difference on the overall QoL scale, which is in line with the current study [60, 75–78]. This is confirmed by the systematic review of Mols et al., stating that long-term breast cancer survivors experience good overall QoL but face specific functional and symptom-related problems [79]. An explanation for the overall good QoL among AYA breast cancer survivors can be the posttraumatic growth that cancer survivors develop after surviving cancer. Post-traumatic growth can result in a more positive outlook on life and is thought to improve one’s perception of overall QoL despite the ongoing functional and symptom-related challenges [80, 81].

### Clinical implications

It is valuable to discuss these long-term HRQoL effects with AYA breast cancer survivors during consultations and treatment decisions. Although novel treatments have enhanced breast cancer survival rates over the years, they also have negative effects [82]. Understanding these impacts can improve shared decision-making, helping patients and healthcare providers weigh the benefits and potential long-term consequences of treatment options.

Additionally, the results of the current study emphasize the importance of tailored follow-up care for AYA breast cancer survivors. While the direct supportive care for AYA cancer survivors has improved, there is a need for more comprehensive long-term support to address persistent physical and psychosocial challenges [8]. Factors such as a negative body image, maladaptive coping mechanism, unemployment and persistent fatigue, insomnia, and pain can be used to identify patients at risk for lower long-term HRQoL outcomes and are essential for effectively targeting interventions. Psychological and social interventions or support groups that enhance coping skills (with health problems) and address body image concerns should be components of survivorship care for AYAs, as highlighted by existing literature [8, 83, 84]. Furthermore, a tiered approach is essential to manage resources and ensure timely interventions efficiently. This could range from providing personalized information and accessible self-management tools through digital platforms designed for AYAs to more intensive support like regular checkups and contact with an AYA clinical nurse specialist when certain problems persist or worsen [8, 85, 86].

### Strengths and limitations

To our knowledge, this is one of the first studies examining the long-term HRQoL of AYA breast cancer survivors, providing both healthcare professionals and AYA breast cancer survivors with valuable insights. The comparison with the normative population was essential to determine whether these survivors experience more long-term issues than typically expected, which is vital for understanding the extended impacts of their cancer diagnosis. Additionally, the comparison with the normative population enabled adjustment for the background risk of HRQoL impairments. Another key strength of this study is the availability of long-term HRQoL data and the large sample size (n = 944), which enhances the reliability of the results [3]. Moreover, the wide variety of demographic and clinical data within the SURVAYA database and the NCR allowed for the exploration of various potential associations. Furthermore, the non-responder analysis demonstrated no significant differences in baseline characteristics, which reduces the risk of selection bias.

A limitation of this study is the lack of data on several factors that can influence HRQoL. Firstly, data concerning the type of breast reconstruction was unavailable, as only primary breast surgeries were recorded within the NCR. Previous literature demonstrated that receiving a breast reconstruction has a significant positive influence on HRQoL [87]. Additionally, detailed data regarding the type and duration of hormonal therapy was missing. Breast cancer patients often undergo hormonal treatments for several years post-diagnosis (for AYA patients, often up to 10 years) to reduce the risk of recurrence. These treatments can have significant negative effects on HRQoL due to side effects such as sweating, hot flashes, and vaginal dryness. Moreover, different types of hormonal therapies, including ovarian function suppression, can have varying impacts on HRQoL [48, 49, 88–91]. Furthermore, data on menopausal status was absent. The systemic treatments that AYA breast cancer survivors receive can lead to early menopause and ovarian dysfunction, significantly affecting HRQoL [92]. Lastly, data on recurrence or distant metastasis was missing. Recurrence or distant metastasis can impact HRQoL, as they indicate a poorer prognosis and necessitate more aggressive treatments [93]. Future research should aim to include these factors to understand their distinct effects on patient outcomes. Another limitation is that only body image, which was associated with all scales of the EORTC QLQ-C30, and coping mechanisms were included as personality traits in the current study. A recent systematic review found that more personality traits such as optimism and neuroticism play an important role in the HRQoL of breast cancer patients [94]. Future research should aim to consider additional personality traits to interpret long-term HRQoL outcomes more accurately. Additionally, the assessment of cognitive functioning and pain is another limitation, as it relies on only a few questions from the EORTC QLQ-C30. Given that these scales showed large significant differences compared to the normative population, a more comprehensive examination in future studies would be valuable (e.g., issues with memory, concentration, processing speeds, and types of pain like surgical, radiative, nerve-related, or hormonal therapy-induced). Lastly, the AYA breast cancer survivors included in the current study were treated in academic hospitals and the NCI, which likely represents a different population compared to AYA breast cancer survivors treated in general hospitals, which may limit the generalizability of the results.

## Conclusion

AYA breast cancer survivors have significantly lower HRQoL, with decreased functioning and a greater burden of specific symptoms, compared to the matched normative population 5–20 years after their diagnosis. Age, BMI, relationship status, educational level, employment status, chemotherapy, body image, and coping mechanisms are all significantly associated with long-term HRQoL. The results of this study highlight the need for improvement in follow-up care and long-term support to enhance the HRQoL of AYA breast cancer survivors in the future. Furthermore, it is valuable to consider these long-term HRQoL effects during shared decision-making to support patient well-being and treatment satisfaction better.

## Electronic supplementary material

Below is the link to the electronic supplementary material.

Supplementary file1 (DOCX 35 KB)

## Data Availability

The data presented in this study are available on request from the corresponding author. The data are not publicly available due to privacy issues.

## Appendix

See Tables

**Table 4:** Baseline characteristics of the normative population

Characteristics	Overall (N = 409)
Age at time of filling out EORTC QLQ-C30 (years)	
Mean (SD)	47.6 (9.11)
Median [Min, Max]	47.0 [21.00, 64.00]
Relationship status, n (%)	
Married	234 (57.2)
Divorced	56 (13.7)
Widowed	13 (3.2)
Single	106 (25.9)
Educational level, n (%)	
Primary school	17 (4.2)
Pre-vocational secondary education	75 (18.3)
Senior general secondary education/pre-university education	37 (9.0)
Secondary vocational education	132 (32.3)
Higher professional education	100 (24.4)
University	48 (11.7)

*EORTC QLQ-C30* European Organization for Research and Treatment of Cancer Quality of Life Questionnaire Core-30, *SD* standard deviation

4,

**Table 5:** Non-responder analysis

Characteristics	Responder (n = 887)	Non-responder (n = 57)	P-value
Age at diagnosis (years)			
Mean (SD)	34.8 (3.84)	34.6 (3.99)	0.75
Median [Min, Max]	36.0 [18.0, 39.0]	36.0 [25.0, 39.0]	
Time since diagnosis (years)			
Mean (SD)	12.2 (4.49)	12.7 (4.87)	0.441
Median [Min, Max]	12.2 [4.41, 21.6]	13.6 [5.05, 20.2]	
Age at time of filling out EORTC QLQ-C30 (years)			
Mean (SD)	47.4 (6.16)	47.8 (5.77)	0.675
Median [Min, Max]	47.8 [23.7, 60.0]	18.1 [32.5, 58.9]	
BMI, n (%)			
Normal weight	547 (61.7)	26 (45.6)	0.825
Overweight	336 (37.9)	18 (31.6)	
Missing	4 (0.5)	13 (22.8)	
Relationship status, n (%)			
Registered partnership/married	509 (57.4)	36 (63.2)	0.604
Relationship	235 (26.5)	12 (21.1)	
Single	139 (15.7)	8 (14.0)	
Missing	4 (0.5)	1 (1.8)	
Educational level, n (%)			
Low, no primary school	4 (0.5)	1 (1.8)	0.329
Intermediate, secondary education	352 (39.7)	25 (43.9)	
High, college/university	531 (59.9)	31 (54.4)	
Employment status, n (%)			
Employed	629 (70.9)	35 (61.4)	0.285
Self employed	118 (13.3)	11 (19.3)	
Unemployed	140 (15.8)	11 (19.3)	
Tumor stage, n (%)			
1	320 (36.1)	18 (31.6)	0.822
2	418 (47.1)	29 (50.9)	
3	143 (16.1)	10 (17.5)	
4	6 (0.7)	0 (0)	
Chemotherapy, n (%)			
Yes	756 (85.2)	51 (89.5)	0.492
No	131 (14.8)	6 (10.5)	
Radiotherapy, n (%)			
Yes	683 (77.0)	47 (82.5)	0.429
No	204 (23.0)	10 (17.5)	
Hormonal therapy, n (%)			
Yes	452 (51.0)	27 (47.4)	0.697
No	435 (49.0)	30 (52.6)	
Targeted therapy, n (%)			
Yes	165 (18.6)	8 (14.0)	0.492
No	722 (81.4)	49 (86.0)	
Breast and axillary surgery, n (%)			
BCS	346 (39.0)	23 (40.4)	0.971
BCS + ALND	124 (14.0)	9 (15.8)	
Mastectomy	204 (23.0)	12 (21.1)	
Mastectomy + ALND	206 (23.2)	13 (22.8)	
Missing	7 (0.8)	0 (0)	

*BCS* breast-conserving surgery, *ALND* axillary lymph node dissection, *BMI* body mass index, *EORTC QLQ-C30* European Organization for Research and Treatment of Cancer Quality of Life Questionnaire Core-30, *SD* standard deviation

5 and

**Table 6:** EORTC QLQ-C30 scores of AYA breast cancer survivors compared to the normative population

	AYA breast cancer survivors (n = 887)	Normative population (n = 409)	P-value
*Functional scales (0–100)*			
QoL			
Mean (SD)	75.2 (17.7)	75.2 (19.5)	0.405
Median [Min, Max]	75.0 [8.33, 100]	83.3 [0, 100]	
Missing, *n* (%)	8 (0.9%)	1 (0.2%)	
Physical functioning			
Mean (SD)	90.0 (13.6)	91.9 (13.7)	** < 0.001**
Median [Min, Max]	93.3 [0, 100]	100 [33.3, 100]	
Missing, *n* (%)	1 (0.1%)	0 (0%)	
Role functioning			
Mean (SD)	80.7 (26.1)	88.7 (22.0)	** < 0.001**
Median [Min, Max]	100 [0, 100]	100 [0, 100]	
Missing, *n* (%)	4 (0.5%)	0 (0%)	
Emotional functioning			
Mean (SD)	76.8 (21.0)	82.8 (20.4)	** < 0.001**
Median [Min, Max]	83.3 [0, 100]	91.7 [0, 100]	
Missing, *n* (%)	2 (0.2%)	1 (0.2%)	
Cognitive functioning			
Mean (SD)	72.4 (25.3)	91.4 (16.5)	** < 0.001**
Median [Min, Max]	83.3 [0, 100]	100 [0, 100]	
Missing, *n* (%)	2 (0.2%)	1 (0.2%)	
Social functioning			
Mean (SD)	86.6 (22.9)	92.0 (17.8)	** < 0.001**
Median [Min, Max]	100 [0, 100]	100 [0, 100]	
Missing, *n* (%)	5 (0.6%)	1 (0.2%)	
*Symptom scales (0–100)*			
Fatigue			
Mean (SD)	32.8 (25.3)	20.1 (23.1)	** < 0.001**
Median [Min, Max]	33.3 [0, 100]	11.1 [0, 100]	
Missing, *n* (%)	2 (0.2%)	0 (0%)	
Nausea and vomiting			
Mean (SD)	3.80 (10.3)	4.65 (12.1)	0.626
Median [Min, Max]	0 [0, 100]	0 [0, 83.3]	
Missing, *n* (%)	2 (0.2%)	0 (0%)	
Pain			
Mean (SD)	20.0 (24.4)	15.8 (23.7)	** < 0.001**
Median [Min, Max]	16.7 [0, 100]	0 [0, 100]	
Missing, *n* (%)	1 (0.1%)	0 (0%)	
Dyspnea			
Mean (SD)	8.10 (16.9)	5.38 (13.9)	**0.004**
Median [Min, Max]	0 [0, 100]	0 [0, 100]	
Missing, *n* (%)	2 (0.2%)	0 (0%)	
Insomnia			
Mean (SD)	29.2 (29.8)	20.4 (27.7)	** < 0.001**
Median [Min, Max]	33.3 [0, 100]	0 [0, 100]	
Missing, *n* (%)	3 (0.3%)	0 (0%)	
Appetite loss			
Mean (SD)	6.21 (17.0)	4.81 (13.7)	0.299
Median [Min, Max]	0 [0, 100]	0 [0, 66.7]	
Missing, *n* (%)	2 (0.2%)	0 (0%)	
Constipation			
Mean (SD)	7.83 (18.5)	6.29 (15.2)	0.379
Median [Min, Max]	0 [0, 100]	0 [0, 100]	
Missing, *n* (%)	2 (0.2%)	1 (0.2%)	
Diarrhea			
Mean (SD)	4.91 (14.2)	4.82 (13.7)	0.94
Median [Min, Max]	0 [0, 100]	0 [0, 66.7]	
Missing, *n* (%)	4 (0.5%)	1 (0.2%)	
Financial impact			
Mean (SD)	12.9 (25.8)	4.49 (15.1)	** < 0.001**
Median [Min, Max]	0 [0, 100]	0 [0, 100]	
Missing, *n* (%)	5 (0.6%)	1 (0.2%)	

*AYA* Adolescent and Young Adult, *QoL* quality of life, *SD* standard deviation

Bold indicates P < 0.05

^6^.
